# Outside-In Anterior Cruciate Ligament Revision With Lateral Tenodesis and High-Strength Suture Augmentation Is Easy to Perform With the Iliotibial Band

**DOI:** 10.1016/j.eats.2021.01.032

**Published:** 2021-04-18

**Authors:** Vincent Marot, Arnault Valette, Louis Courtot, Thibault Lucena, Nicolas Reina, Etienne Cavaignac

**Affiliations:** a Musculoskeletal Institute, Hôpital Pierre Paul Riquet, Centre Hospitalier Universitaire de Toulouse, Toulouse, France; b Orthopaedics Unit, Hospital Nostra Senyora de Meritxell, Escaldes-Engordany, Andorra; c I2R, Institut de Recherche Riquet, Toulouse, France; d SPS Research, Toulouse, France

## Abstract

We describe a technique for revision anterior cruciate ligament (ACL) surgery using a 15-cm strip of the iliotibial band as a graft and the gracilis tendon if available. An internal brace is added to augment the graft. The graft is passed through the femur by drilling an outside-in tunnel from the isometric point F9 of Krackow toward the ACL’s footprint and is then double fixed at the tibia using an interference screw and a cortical button. This technique makes it possible to perform simultaneous ACL reconstruction and lateral tenodesis with a continuous, rigid, good-diameter graft that is pedicled to the Gerdy tubercle. Good rotational control is achieved, and all the factors that contribute to ligamentization are present.

The retear rate after anterior cruciate ligament (ACL) reconstruction is substantial, ranging from 4.4%^1^ to 20% in the youngest patients.^2^ The incidence of a second failed ACL reconstruction is not reported in the literature. Additional reconstruction procedures are rarely performed, given the doubts about the benefits of a new surgical procedure after 2 failures and because of increased risks of osteoarthritis.^3^

The fascia lata or iliotibial band (ITB) is biomechanically suitable as an ACL graft. According to Chan et al.,^4^ the initial tensile strength (3,266 N) and stiffness are equal to or greater than those of several other candidate ACL graft tissues, including the patellar tendon. If the gracilis is present, adding this autograft tendon increases the diameter or length of the ITB graft when it is too short or too thin.^5^

Our purpose is to describe a technique for arthroscopic revision after an ACL retear using an ITB autograft, augmented by a gracilis autograft if present and an internal brace.

## Surgical Technique

### Preoperative Assessment

**Fig 1:**
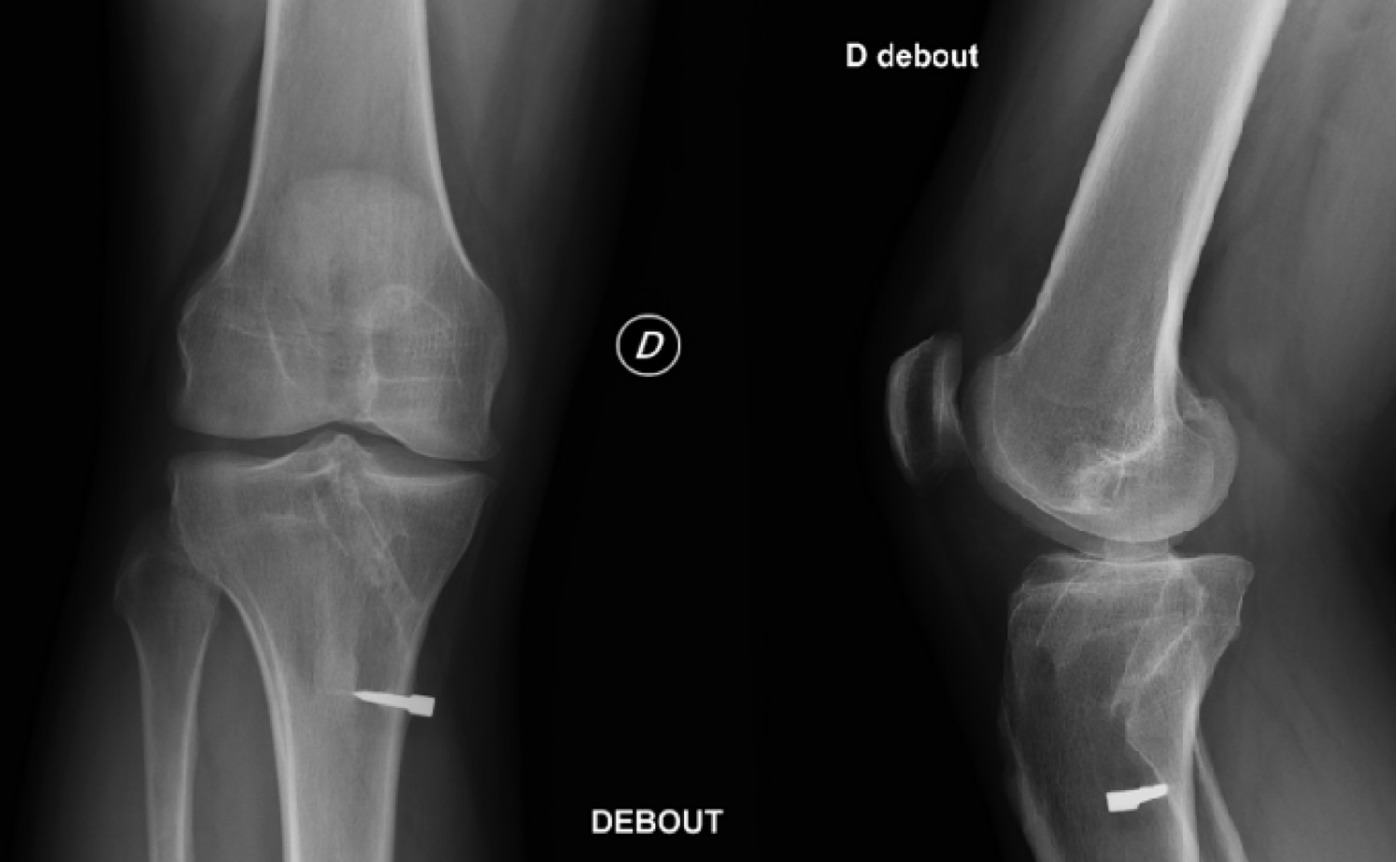
Preoperative radiographs of right (D) knee. The patient underwent prior anterior cruciate ligament and posterior cruciate ligament reconstruction surgery.

**Fig 2:**
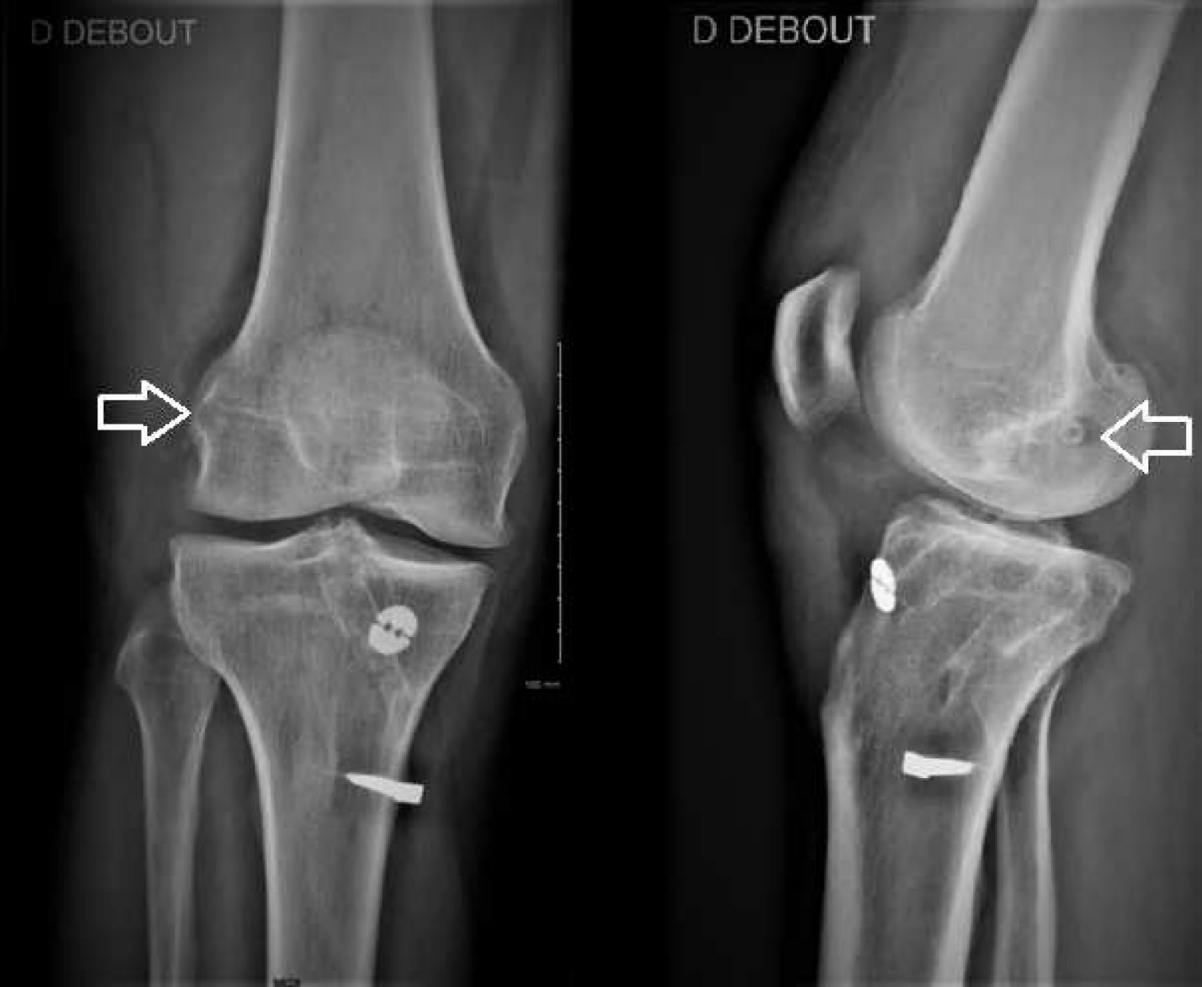
Postoperative radiographs of right (D) knee. We plan to drill the femoral tunnel with an outside-in technique, which minimizes the risk of tunnel overlap and, thereby, 2-stage revision. In this knee, we can see that the new femoral tunnel (arrows) is behind the lateral femoral condyle. There is no convergence between the existing and new femoral tunnels.

The causes of the retear must be identified among the following: trauma (32%), technical error (improper femoral and tibial tunnel placement, 24%), biological (7%), or a combination of the aforementioned factors (37%).^6^ Bony malalignment must also be evaluated: posterior tibial slope greater than 12° and varus malalignment increase the stress on the ACL. Osteotomies can be performed in conjunction with the revision. A computed tomography scan is obtained preoperatively to evaluate the placement and diameter of the existing tunnels. Single-stage ACL revision can be performed in patients who have appropriately positioned tunnels. If the existing tunnels will be used, the tunnels must be measured to ensure that their diameter does not exceed 15 mm.^7^ Two-stage ACL revision reconstruction, with bone grafting, should be performed when tunnel osteolysis is present or if the existing tunnels will overlap with the new tunnels. We prefer to drill the femoral tunnel using an outside-in technique, which limits the risk of tunnel overlap and, thus, of 2-stage revision (Figs 1 and 2).

### Patient Positioning

**Fig 3:**
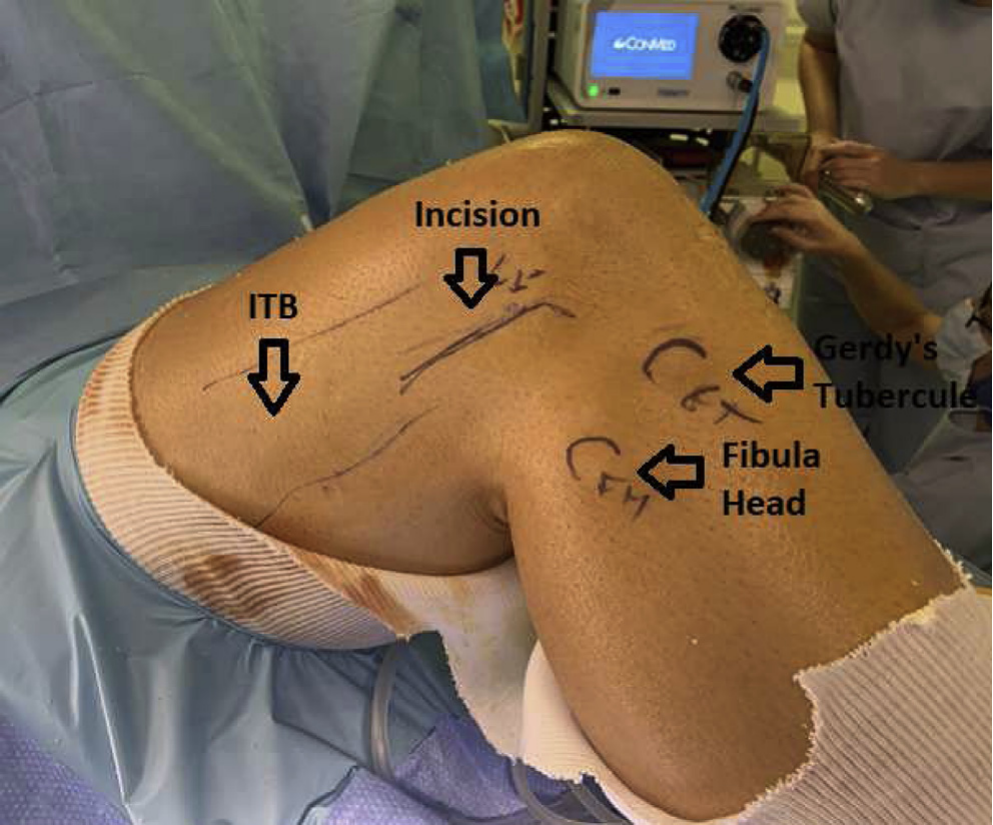
Lateral view of patient's flexed right knee positioning for outside-in anterior cruciate ligament revision with lateral tenodesis and high-strength suture augmentation. The patient is placed supine on the operating table in the standard arthroscopy position. A tourniquet is applied at the upper thigh. A lateral post proximal to the knee is positioned at the level of the tourniquet, in addition to 2 foot rolls at 90° and 120° of flexion. A right knee is shown with arrows indicating the locations of the iliotibial band (ITB), Gerdy tubercle, and fibula head, as well as the incision for graft harvesting.

The patient is placed supine on the operating table in the standard arthroscopy position (Video 1). A tourniquet is applied at the upper thigh. A lateral post proximal to the knee is positioned at the level of the tourniquet, in addition to 2 foot rolls at 90° and 120° of flexion (Fig 3).

### Graft Harvesting

##### Harvesting of ITB

**Fig 4:**
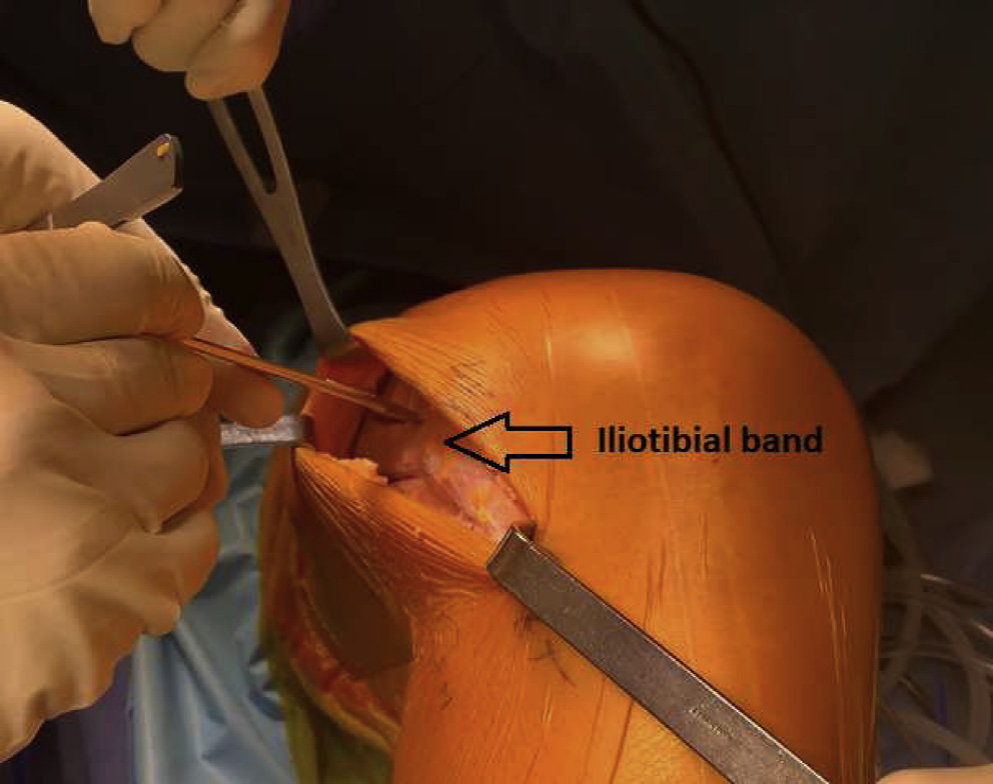
Anterolateral view of right knee showing iliotibial band graft harvesting. A 10-cm skin incision is made 2 cm proximal to the Gerdy tubercle. The posterior ridge from the Gerdy tubercle is incised with a No. 23 blade and extended proximally 5 to 6 cm; a second incision is made 1 cm anterior and parallel to the previous incision, so the graft is 1 cm wide at its distal end. The proximal portion of the iliotibial band is exposed with 2 retractors, and the posterior incision is extended proximally with the same blade to obtain a total graft length of approximately 15 cm. The anterior incision is then extended to obtain a 3-cm-wide graft at its proximal end.

**Fig 5:**
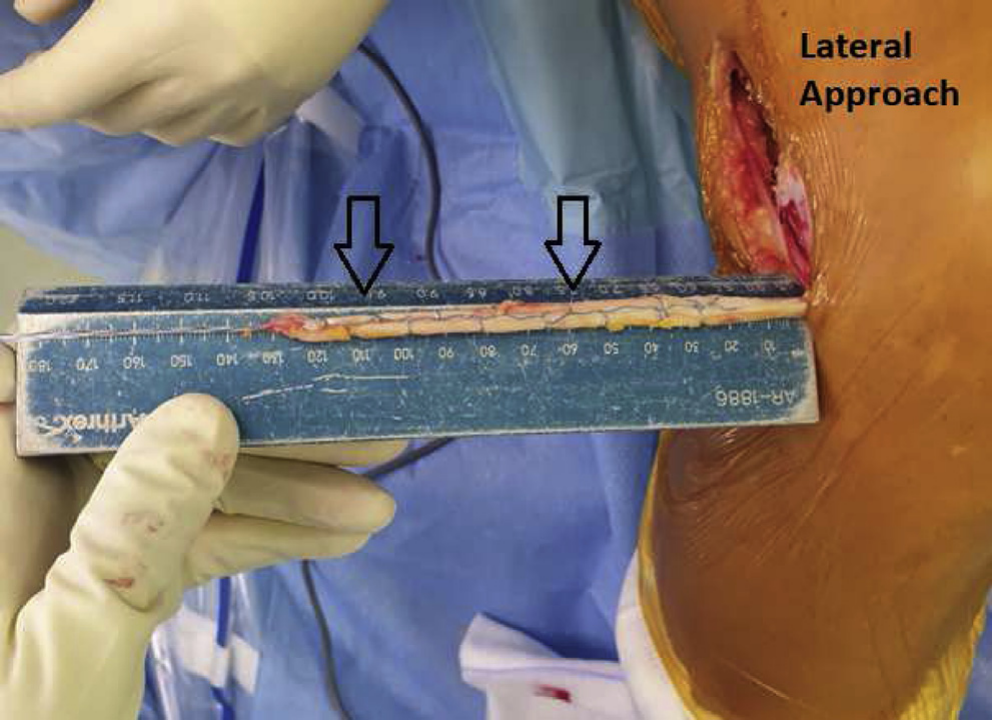
Iliotibial band graft “tubulization” (arrows) made with internal brace (SutureTape) in right knee.

A 10-cm skin incision is made 2 cm proximal to the Gerdy tubercle (GT). The posterior ridge from the GT is incised with a No. 23 blade and extended proximally 5 to 6 cm; a second incision is made 1 cm anterior and parallel to the previous incision, so the graft is 1 cm wide at its distal end. The proximal portion of the ITB is exposed with 2 retractors, and the posterior incision is extended proximally with the same blade to obtain a total graft length of approximately 15 cm. The anterior incision is then extended to obtain a 3-cm-wide graft at its proximal end (Fig 4). The distal end of the ITB is separated from its deep fibers with electrocautery and kept attached to its insertion on the GT. The fat pad is cleared from the graft, and “tubulization”^5^ is performed with an internal brace (SutureTape; Arthrex) (Fig 5). The graft’s diameter is measured. The graft is then wrapped in a compress soaked in vancomycin.

##### Harvesting of Gracilis

The surgeon must confirm that the gracilis was not used during the first ACL reconstruction. A standard vertical 2-cm incision is made medially to the anterior tibial tuberosity. The gracilis tendon is harvested with an open tendon stripper and then cleaned and cut close to its tibial insertion. Hyperflexion provides better access to the most proximal vincula. Tubulization of the ITB is performed around the gracilis tendon in this scenario.

### ACL Reconstruction

**Fig 6:**
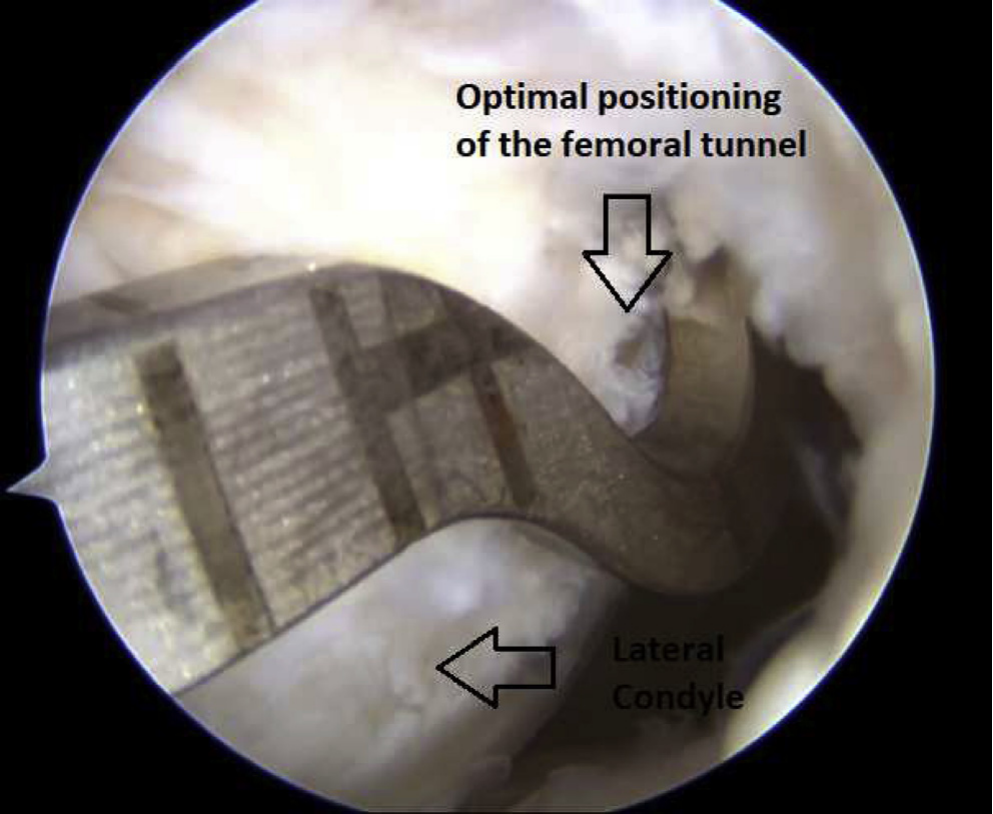
The scope is placed in the anteromedial portal, which ensures the optimal positioning of the intra-articular portion of the femoral tunnel. The medial and posterior sections of the right lateral femoral condyle are shown.

**Fig 7:**
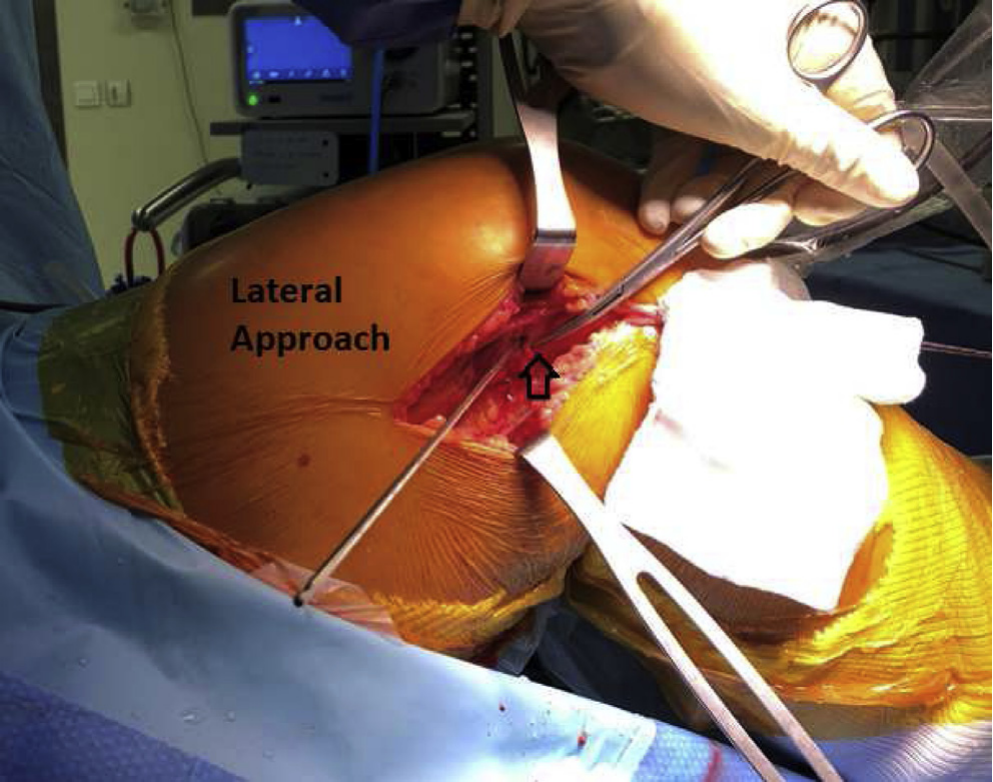
Lateral view of right knee showing K-wire at isometric point F9 of Krackow. The isometric point F9 of Krackow is located 1 cm proximal and slightly posterior to the lateral femoral condyle (arrow) and marked with an electrocautery tip.

**Fig 8:**
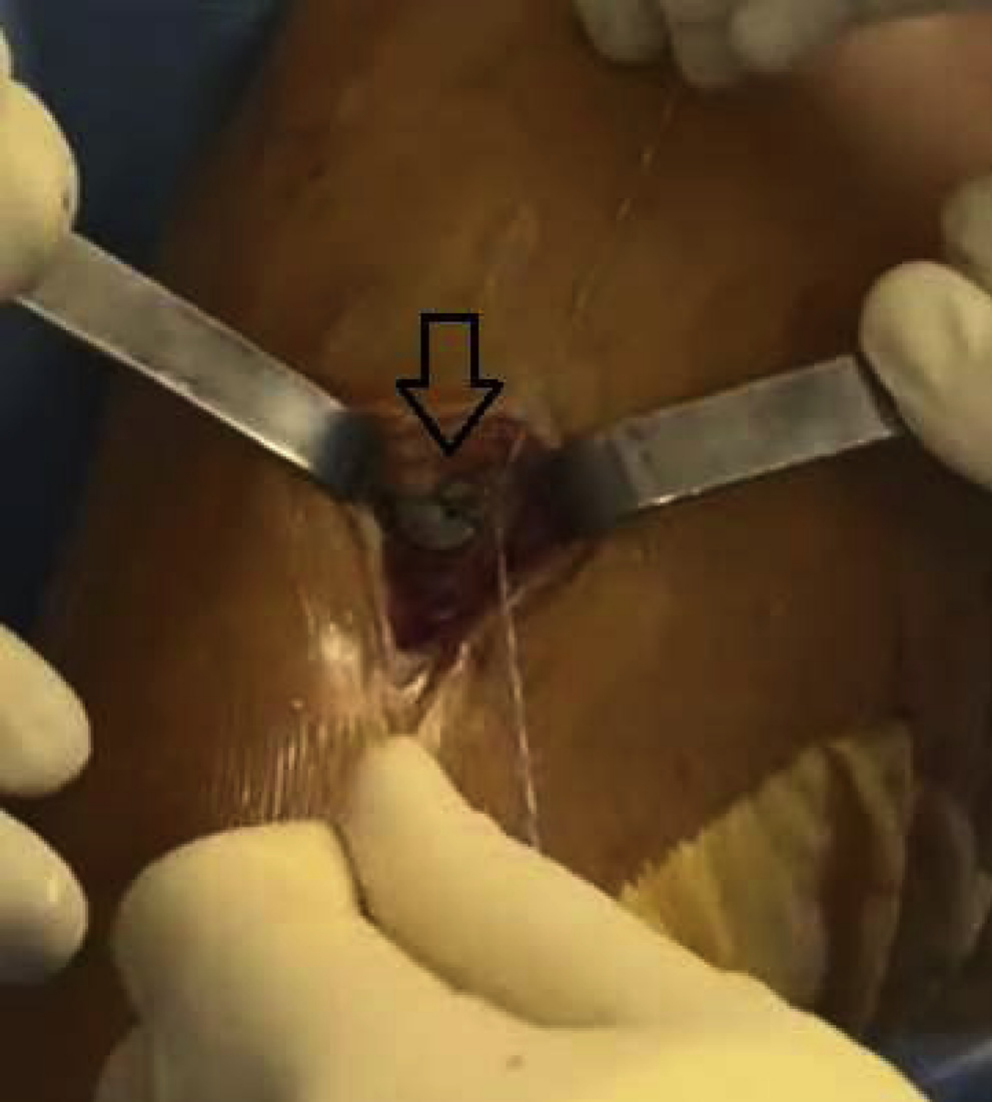
Anteromedial view of right knee. A second tibial fixation point is added by tying the FiberTape around a TightRope ABS implant (arrow) on the anteromedial cortex of the right tibia.

The isometric point F9 of Krackow^8^ is located 1 cm proximal and slightly posterior to the lateral femoral condyle and marked with an electrocautery tip. An outside-in ACL femoral guide (Arthrex) is introduced through the anterolateral portal, inserted at the femoral footprint of the ACL. The scope is placed in the anteromedial portal, which ensures the optimal positioning of the intra-articular portion of the femoral tunnel (Fig 6). The guide’s angulation is then adjusted to allow drill sleeve placement in the stab incision at the level of the previously identified isometric point (Fig 7). After guide pin placement, a femoral tunnel of the same diameter as the graft is drilled over 5 mm. A tibial tunnel of the same diameter as the graft is drilled over 5 mm, completely from the hamstring incision, with an outside-in guide set at 55° to 60° (Arthrex), potentially through the existing tunnel if it is in a good position. The relay suture is passed through the tibial tunnel and then via the femoral tunnel from inside to outside using a FiberStick (Arthrex). The free limbs of the SutureTape are retrieved at the opening of the tibial tunnel. The graft is passed proximal to distal and then secured under tension in 30° of flexion using 2 BioComposite interference screws (Arthrex). One screw secures the graft to the femur, from outside in, while we ensure that the screw does not stick out, because it could rub against the fascia lata postoperatively. The second screw is used to secure the graft to the tibia. A second tibial fixation point is added by tying the FiberTape (Arthrex) around a TightRope ABS implant (Arthrex) on the anteromedial cortex of the tibia (Fig 8).

### Wound Closure

**Fig 9:**
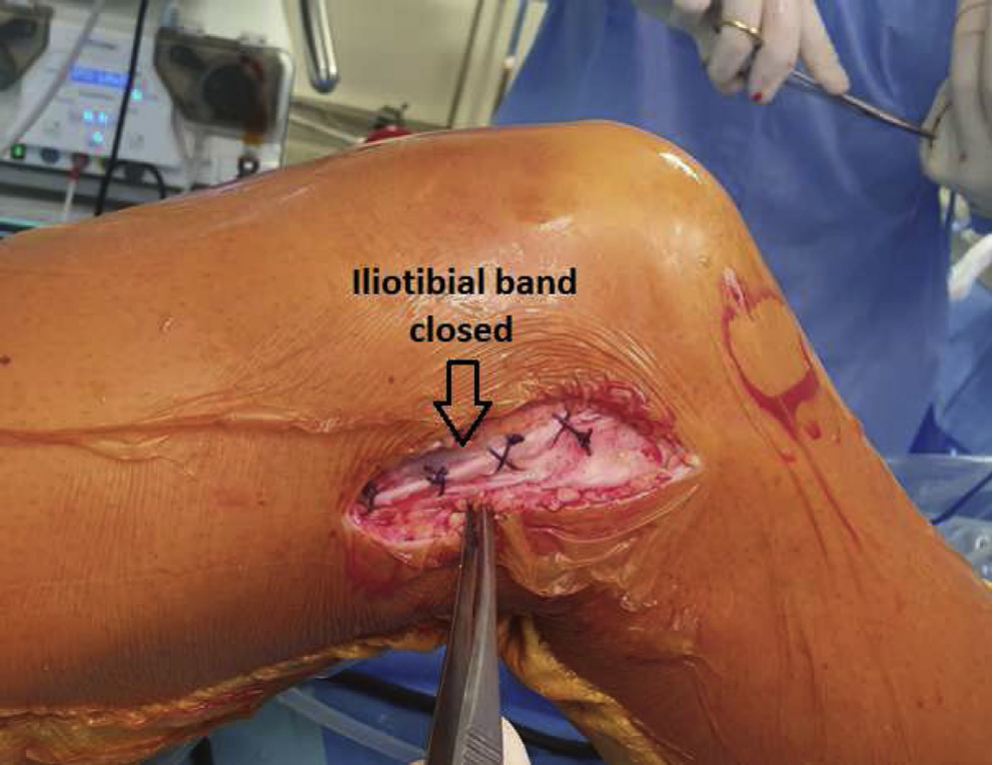
Lateral view of right knee. At the end of the procedure, the iliotibial band is closed with absorbable No. 2 Vicryl sutures.

The tourniquet is deflated, and meticulous hemostasis is ensured, especially bleeding from the vessels deep to the ITB, just behind the lateral condyle. We recommend using the intermuscular septum sectioning technique, which enables posterior fascia advancement.^9^ The ITB is closed with absorbable No. 2 Vicryl sutures (Ethicon) (Fig 9). Subcutaneous and skin layers are closed with an absorbable suture. Pearls and pitfalls for performing the described surgical procedure are listed in Table 1.

**Table 1:** Pearls, Pitfalls, and Risks

Pearls
The graft is 1 cm wide at its distal part and 3 cm at its proximal part.
A total graft length ≥ 15 cm is necessary.
If the gracilis is present, it can be harvested and added to the ITB graft if the latter is too short or too thin.
The graft is braided with SutureTape.
The femoral tunnel is drilled with an outside-in technique at the isometric point F9 of Krackow.
Double tibial fixation using an ABS cortical button (Arthrex) is possible.
Sectioning the intermuscular septum allows for posterior fascia advancement, which makes it easier to close the ITB.
Pitfalls and risks
There is a risk of muscle hernia proximally where the ITB graft was harvested; for this reason, the fascia must be closed meticulously.
There is a risk of hematoma on the lateral part of the thigh; for this reason, the tourniquet must be released and any bleeding must be stopped before closing.
There is a risk of iatrogenic injury to the LCL when drilling the femoral tunnel; this is easily avoidable by palpating and marking the LCL before drilling.

ITB, iliotibial band; LCL, lateral collateral ligament.

### Postoperative Rehabilitation

The knee is not immobilized with a brace, except in cases with radial or complete meniscal root tears. All patients undergo surgery on an outpatient basis. A routine ACL rehabilitation program is started on the first postoperative day, entailing full weight bearing and progressive exercises to regain range of motion and quadriceps function. A gradual return to sports is generally allowed starting at 4 months for nonpivoting sports, 6 months for noncontact pivoting sports, and 8 to 9 months for contact pivoting sports, after isokinetic tests and a functional evaluation.

## Discussion

The described ACL revision technique, which uses the ITB and gracilis as grafts and is augmented by an internal brace, has several advantages: (1) ACL reconstruction and lateral tenodesis can be performed simultaneously. Lateral tenodesis is an effective supplement for added rotational control in the setting of ACL revision.^10^ Lateral tenodesis also reduces the loads on the ACL, which in turn reduces the risk of another retear. (2) The ITB graft’s distal attachment is left intact, and the graft is continuous. According to several studies performed on hamstring tendons, this ensures that the vascular network is maintained and may contribute to maturation and ligamentization of the graft.^11^ We can easily imagine that the same processes occur with the ITB. (3) Drilling the femoral tunnel using an outside-in technique limits the risk of tunnel overlap and, thus, of 2-stage revision. The tunnels drilled during the revision may be in different positions than the existing tunnels, even if convergence occurs at the opening of the tunnels, as long as the graft fixation is not compromised. (4) Performing a single-stage revision saves time and avoids the risks, morbidity, and costs of a second procedure for the patient, along with a second round of postoperative physical therapy sessions.

The gracilis is not essential to this ACL revision technique; however, if it is still intact, harvesting it adds to the graft’s diameter or length when the ITB is too short or too thin.^5^ In fact, certain studies have shown that the risk of retear is reduced by 0.82 to 0.86 each time the graft’s diameter increases by 0.5 mm.^12^ Even though ITB grafts are often small in size, the risk of retear is minimized by the fact that this technique combines lateral tenodesis and ACL reconstruction during the same surgical session. This also makes it possible to drill smaller-diameter tunnels, which is better in revision scenarios because of the risk of overlapping with the existing tunnels. Thus, gracilis harvesting is optional and is performed only when the ITB graft is inadequate.

One of the challenges of ACL revision is having to drill a new femoral tunnel. Most primary ACL reconstructions use an inside-out technique (anteromedial or transtibial). Attaching the graft is more difficult when the tunnel drilled for the previous reconstruction is large or has widened, which may require a 2-stage revision and bone grafting.^13^ Using an outside-in femoral tunnel makes it easier to drill the new tunnel by limiting the risk of overlap, controlling the intra- and extra-articular exit points, and reducing the risk of posterior cortical collapse.^14^ In addition, according to Hiramatsu et al.,^15^ the outside-in technique yields a more acute femoral graft bending angle, longer mean femoral tunnel length, and larger contact ratio than the inside-out technique. Finally, the outside-in femoral tunnel drilling technique can be used in pediatric patients because it does not entail passage through the femoral growth plate.^16^

Our technique uses an internal brace (FiberTape) to increase the graft’s rigidity and protect it until the end of the ligamentization process. One study has shown that an internal brace increases the biomechanical performance of intra-articular ligament reconstructions and does not affect bone tunnel healing.^17^ Using FiberTape also makes it possible to add a second tibial fixation point on the anteromedial cortex of the tibia with a TightRope ABS device. The advantages and limitations of this surgical technique are listed in Table 2.

**Table 2:** Advantages and Limitations

Advantages
The technique has low donor-site morbidity and does not alter the quadriceps or hamstring tendons.
Simultaneous ACL reconstruction and lateral tenodesis can be performed.
Graft remains attached to the Gerdy tubercle and is continuous, which may contribute to ligamentization.
Drilling an outside-in femoral tunnel makes it easy to create the new tunnel by limiting the risk of overlap, controlling the intra- and extra-articular exit points, and reducing the risk of posterior cortical collapse.
Augmentation by an internal brace (FiberTape) increases the graft’s rigidity and provides a second tibial fixation point.
Limitations
Larger scars occur than with other types of ACL revision graft.

ACL, anterior cruciate ligament.

Our ACL revision technique combining ITB and gracilis autografts augmented by an internal brace is simple and easy to perform. We have performed more than 50 ACL revisions using the ITB and gracilis in the past 2 years and have not seen any retears. A randomized controlled trial comparing our technique and a technique combining a bone–patellar tendon–bone graft and modified Lemaire lateral tenodesis is underway. The early results are encouraging (L. Courtot, unpublished data, March 2020).

## Supplementary Data

Video 1 Overview of outside-in anterior cruciate ligament revision technique with iliotibial band. First, we describe the main anatomic landmarks; then, we describe the technique used to harvest the graft, to create the tunnel, and to fix the graft. The video emphasizes tips and tricks useful for this surgical procedure such as the intermuscular septum sectioning technique, which enables posterior fascia advancement.

ICMJE author disclosure forms
